# Sex-specific association between triglyceride-glucose index and all-cause mortality in patients with osteoporotic fractures: a retrospective cohort study

**DOI:** 10.3389/fendo.2025.1574238

**Published:** 2025-04-30

**Authors:** Shao-han Guo, Jian Xu, Ya-qin Gong, Wen-bin Hu, Chong Li, Ke Lu

**Affiliations:** ^1^ Department of Orthopedics, Affiliated Kunshan Hospital of Jiangsu University, Suzhou, Jiangsu, China; ^2^ Kunshan Biomedical Big Data Innovation Application Laboratory, Suzhou, Jiangsu, China; ^3^ Department of Orthopedics, The First People’s Hospital of Kunshan, Gusu School, Nanjing Medical University, Suzhou, Jiangsu, China; ^4^ Information Department, Affiliated Kunshan Hospital of Jiangsu University, Suzhou, Jiangsu, China; ^5^ Chronic Disease Department, Kunshan Center for Disease Control and Prevention, Suzhou, Jiangsu, China

**Keywords:** osteoporotic fracture, all-cause mortality, sex difference, insulin resistance, triglyceride-glucose index, metabolic health

## Abstract

**Background:**

Osteoporotic fractures (OPFs) pose a considerable global health burden and are linked with an elevated mortality risk. The triglyceride-glucose index (TyG-I) is a recognized marker of insulin resistance across various populations. The association between all-cause mortality (ACM) and the TyG-I has been widely investigated in a variety of clinical settings. The potential sex-specific differences in this association among OPF patients remain uncertain.

**Methods:**

In this retrospective cohort study, 2,307 patients ≥ 50 years old admitted to the hospital between January 2018 and August 2023 for surgical treatment of OPFs were included. The TyG-I was determined using fasting triglyceride and glucose levels measured at admission. The association between ACM and the TyG-I was evaluated by Cox proportional hazards regression, adjusting for possible confounding variables. Analyses were categorized by sex, and subgroup analyses evaluated possible interaction effects. The ACM rates among TyG-I tertiles were compared *via* Kaplan–Meier curves.

**Results:**

This research study analyzed 2,307 patients, of whom 247 (10.71%) died from any cause during the follow-up period. In females, a linear association of the TyG-I with ACM was observed even after adjusting for confounders, with each unit increase in the TyG-I correlating with a 37% increased risk of death (HR: 1.37, 95% CI: 1.06-1.77, *p* = 0.02). However, in males, there was a non-linear correlation, where patients in the uppermost TyG-I tertile showed a substantially decreased mortality risk relative to those in the lowest tertile (HR: 0.53, 95% CI: 0.30–0.92, *p =* 0.02). TyG-I indicated a statistically significant relation with sex (*P* for interaction = 0.01).

**Conclusion:**

In patients diagnosed with OPFs, distinct sex-specific variations were observed in the relationship between ACM and the TyG-I. Among female patients, each unit increase in the TyG-I was linked to a 37% greater risk of mortality. Conversely, male patients within the highest TyG-I tertile indicated a lower mortality risk than those in the lowest tertile. Further research is required to confirm these sex-specific associations.

## 1 Introduction

Osteoporotic fractures (OPFs) constitute a critical global health challenge, particularly affecting older populations, with severe implications for mortality, morbidity, and healthcare costs (1). Affecting 8.9 million individuals annually, OPFs are defined by compromised bone strength and vulnerability to minor trauma (2). Mortality after an OPF is a serious issue. Studies have shown that the mortality rate within one year of a hip fracture is between 20% and 30% (3). The function of metabolic factors in the pathogenesis and mortality of OPFs has gained attention, emphasizing further research into relevant metabolic markers (4, 5).

The triglyceride-glucose index (TyG-I) is calculated from fasting glucose and triglyceride (TG) levels and has been proven to be a reliable and cost-effective marker of insulin resistance (IR). It demonstrates higher accuracy compared to traditional methods like the Homeostasis Model Assessment of Insulin Resistance (HOMA-IR) (6). Numerous studies have consistently shown associations between elevated TyG-I and increased all-cause mortality (ACM) in the general healthy population. For instance, Liu et al. observed a significant correlation in a general population cohort (7). However, conflicting evidence exists, as some studies, after controlling for potential confounders, did not observe a similar relationship (8). Heterogeneity in study populations and methodologies may account for these inconsistent findings.

Research on the TyG-I and its relation with ACM has primarily focused on patients with cardiovascular diseases (CVD) or diabetes, leaving its role in OPF populations underexplored. Moreover, sex differences are critical in OPF prevalence and metabolic profile (9, 10). Women, especially those in the post-menopausal phase, have a higher susceptibility to OPFs and show distinct metabolic profiles compared to men. The I-Lan Longitudinal Aging Study highlighted significant sex-specific variations in the association between elevated TyG indices and metabolic outcomes, suggesting that sex may influence the relation between metabolic markers and clinical outcomes (11). These sex-specific variations could influence the association between the TyG-I and mortality risk in OPF patients, thus highlighting the importance of sex-stratified analyses. Therefore, this retrospective cohort study aims to investigate the association between the TyG-I and ACM in OPF patients, focusing on sex-specific differences.

## 2 Materials and methods

### 2.1 Ethical statement

This investigation was approved by the Ethics Committee of the Affiliated Kunshan Hospital of Jiangsu University (AKHJU) under protocol number 2024-03-053-H00-K01 and adhered to the principles of the Declaration of Helsinki. All patient data was de-identified and anonymized to protect patient privacy before analysis. Since the study was observational and the data collection process was anonymous, obtaining written informed consent was not required.

### 2.2 Study population

**Figure 1:**
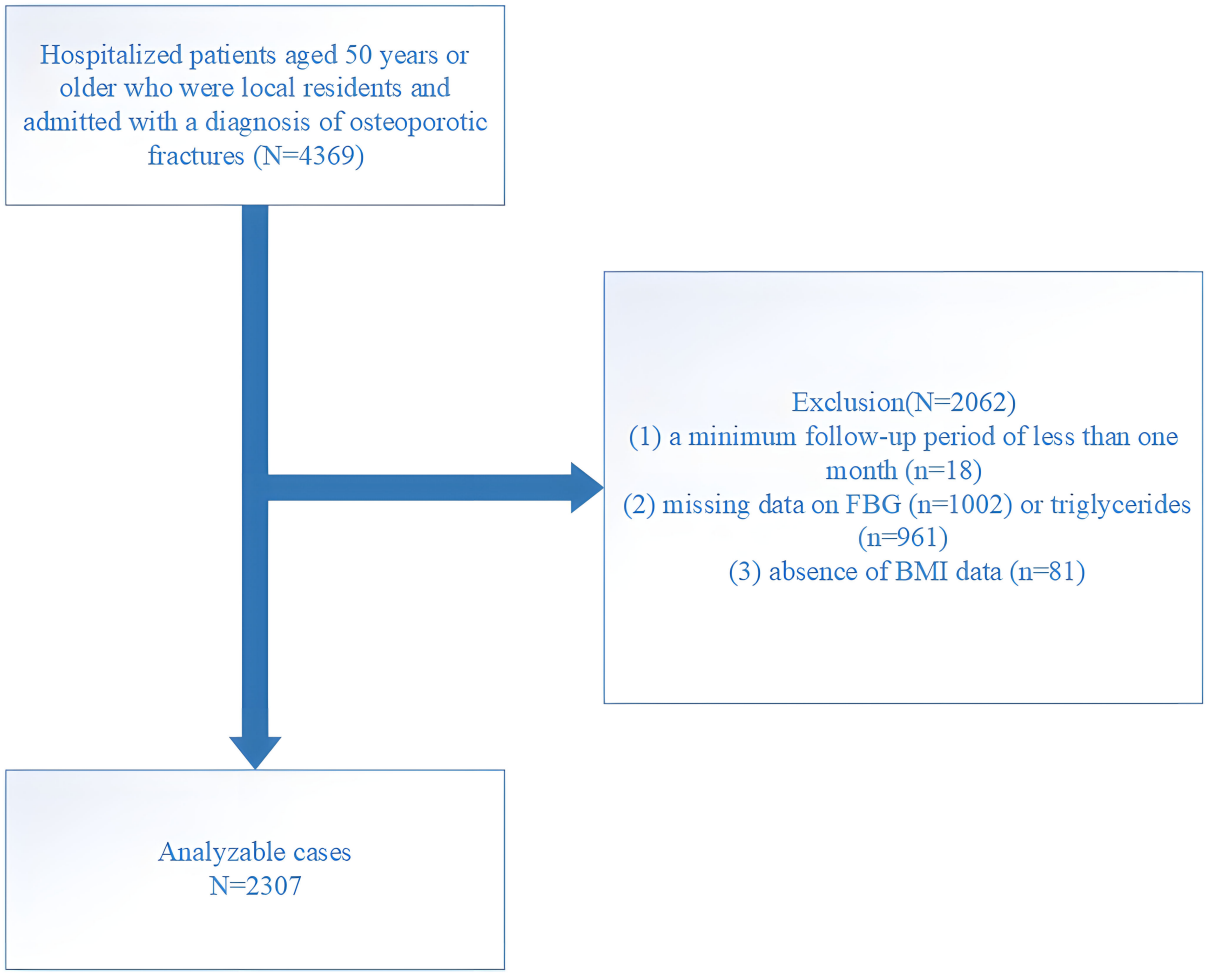
Study flow chart.

This research was conducted at the AKHJU and designed as an open-enrollment retrospective study. Participants included Kunshan residents aged 50 and above who were recently diagnosed with OPF and required immediate hospitalization and surgical treatment. The included participants did not have fractures in the past five years, ensuring the recorded OPF was their first occurrence. The analyzed fractures included the wrist, proximal humerus, hip, and vertebrae (common sites of OPFs, such as the spine, hip, and forearm or humerus fractures, collectively referred to as major OPFs (12)). This research followed the International Statistical Classification of Diseases and Related Health Problems (10^th^ Revision; ICD-10), particularly codes starting with S22, S32, S42, S52, or S72, to identify fractures. The data collection period was from January 1, 2018, to August 25, 2023, with follow-up continuing until April 24, 2024, ensuring at least one month of follow-up for each participant. A total of 4,369 patients were initially selected meeting inclusion criteria, but exclusions were made for the following reasons: (1) follow-up period less than one month (n = 18), (2) missing fasting blood glucose (FBG, n = 1002) or TG data (n = 961), and (3) missing body mass index (BMI) data (n = 81). After these exclusions, 2,307 patients remained in the final cohort (**Figure 1**).

### 2.3 Patient data collection and record

The participants were selected through the Population Death Registration System (PDRS) of Jiangsu Province and the Regional Health Registration Platform (RHRP) of Kunshan City. Follow-up data were gathered systematically by connecting the Affiliated Kunshan Hospital of Jiangsu University Fragility Fracture Registration System (AKHJU FFRS) with the RHRP. This connection relied on patient or hospital IDs, diagnostic details, admission, surgery, and discharge dates (13).

### 2.4 Study exposure variable

The TyG-I was calculated based on FBG and TG levels measured at baseline, in accordance with established methodologies from previous research. The FBG and TG levels were measured within 48 hours of hospital admission (8, 14). The venous blood was sampled after a minimum fasting period of 8 hours and analyzed using an AutoAnalyzer (Beckman-Coulter AU 5800, USA). The TyG-I was evaluated as follows: TyG = Ln [(fasting TG (mg/dL) × FBG (mg/dL))/2].

### 2.5 Study endpoint

The study’s primary outcome was defined as ACM, which is defined as death by any cause. Information on mortality was retrieved from the Jiangsu Province PDRS. Survival time was assessed by evaluating the duration between the discharge date after the initial fracture and the earliest of the following events: death, relocation outside the region, or the study’s conclusion on April 24, 2024.

### 2.6 Covariates

Based on previous studies (15–17), an existing database, and findings from previous clinical research, the following covariates were identified for inclusion in this study: age, body mass index (BMI), blood urea nitrogen (BUN), creatinine (Cr), uric acid (UA), hemoglobin, calcium, high-density lipoprotein (HDL), low-density lipoprotein (LDL), hypertension, diabetes, and fracture type (thoracic vertebra, lumbar vertebra, humerus, radius, femoral neck, femoral trochanteric, and subtrochanteric). BMI was assessed by dividing weight (kg) by the square of height (m²). The Working Group on Obesity in China classifies BMI values of 24–27.9 kg/m² as overweight and values of ≥ 28 kg/m² as obese (18). Blood samples taken at admission were analyzed *via* a Beckman AU5800 biochemistry analyzer (Beckman Coulter, CA, USA). Analytical methods included the uricase-peroxidase protocol for UA, the urease-glutamate dehydrogenase method for BUN, Arsenazo III for calcium, creatininase for Cr, and direct assays for HDL and LDL. Hemoglobin was quantified using a Sysmex XN-10 (B4) hematology analyzer with the SLS method for hemoglobin measurement.

### 2.7 Statistical analyses

Continuous variables were indicated as medians with interquartile ranges (Q1–Q3) or means with standard deviations (SD), while categorical data were depicted as frequencies and percentages (%). The Mann–Whitney U test was carried out for the statistical assessment of non-normally distributed continuous variables, whereas independent two-tailed t-tests were performed for normally distributed variables. The categorical data were elucidated *via* Chi-square tests, with Fisher’s exact test as an alternative when Chi-square assumptions were not met.

The independent association of the TyG-I with ACM was elucidated *via* Cox proportional hazards regression model and adjusted covariance effects. The analysis included three models: Model 1, Model 2, and Model 3. The collinearity diagnostics were evaluated *via* the Variance inflation factor (VIF). Adjustments for covariates were determined based on two criteria. In criterion 1, the basic model was modified, initially limited to ACM and the TyG-I, by removing or adding covariates from/to the full model. The full model included ACM, the TyG-I, and other variables like age, BMI, hemoglobin, calcium, HDL, LDL, Cr, BUN, UA, hypertension, diabetes, and fracture classification. Adjustments aimed to ensure at least a 10% odds ratio (OR) change. Criterion 2 either required meeting Criterion 1 or determining covariates with a < 0.1 *p*-value in univariate analysis (19). Outcomes were compared across Model 1 (unadjusted), Model 2 (adjusted for BMI and age), and Model 3 (further adjusted for Cr, hemoglobin, BMI, calcium, BUN, age, UA, and fracture classification). Since the association of the TyG-I with ACM may vary based on gender, analyses were stratified by sex to explore possible interactions.

The Generalized Additive Model (GAM) was employed to analyze the potential non-linear correlation of the TyG-I with ACM. Two models were compared: Model A, which assumed a linear relationship, and Model B, which accounted for a two-segment nonlinear association. A *p-value* < 0.05 indicated that the nonlinear model better fit the data. Once these relationships were identified, a two-piecewise linear regression model explored threshold effects within the smoothing curves. The maximum likelihood model and a recursive method were employed to autonomously assess the inflection point, particularly when a distinct ratio was observed in the curves (20). Smooth curve fitting was carried out to elucidate the association between ACM and the TyG-I. Survival times for different sex-based groups, stratified by the TyG-I, were evaluated *via* Kaplan–Meier curves. Subgroup analyses were conducted to evaluate the robustness of the findings and identify variations among patient subgroups, stratifying them by specific covariates. Interactions and effect modifications within these subgroups were analyzed using the likelihood ratio test (LRT).

The analysis of data was performed using Empower Stats (X&Y Solutions, Inc., Boston, MA, USA, www.empowerstats.com) and R software (version 4.2.0, http://www.r-project.org). Statistical significance was assessed using two-tailed tests with a threshold of *p* < 0.05.

## 3 Results

### 3.1 Baseline features of two sexes

The study included a total of 2,307 patients. **Table 1** depicts an overview of the baseline features of patients diagnosed with OPF in the study population. Of the total participants, 33.12% (n = 764) were male, and 66.88% (n = 1543) were female. After categorizing female and male patients into three groups based on their TyG-I tertiles, significant differences were observed in biomarkers such as hemoglobin, calcium, HDL, LDL, and UA (*p-value* < 0.01). Moreover, BMI analysis indicated a small but significant increase across the tertiles among female patients (*p-value* < 0.01).

**Table 1:** Characteristics of study participants by TyG-I tertiles across different sexes.

Characteristics	Female				Male			
	Low	Middle	High	*P*-value	Low	Middle	High	*P*-value
N	511	516	516		258	253	253	
Age, mean ± SD, years	69.03 ± 10.51	69.37 ± 10.70	69.45 ± 10.62	0.80	66.55 ± 11.33	68.04 ± 11.58	66.01 ± 11.44	0.12
BMI, mean ± SD, kg/m^2^	22.76 ± 3.34	23.03 ± 3.37	23.42 ± 3.47	<0.01	23.13 ± 3.87	22.90 ± 3.24	23.42 ± 3.48	0.26
Hemoglobin, mean ± SD, g/L	123.65 ± 17.72	123.84 ± 17.71	128.08 ± 16.56	<0.01	122.75 ± 19.74	128.00 ± 18.99	129.93 ± 17.62	<0.01
Calcium, mean ± SD, mmol/L	2.17 ± 0.12	2.20 ± 0.13	2.24 ± 0.14	<0.01	2.17 ± 0.13	2.20 ± 0.12	2.23 ± 0.13	<0.01
HDL, mean ± SD, mmol/L	1.43 ± 0.29	1.33 ± 0.30	1.22 ± 0.31	<0.01	1.45 ± 0.33	1.37 ± 0.29	1.21 ± 0.28	<0.01
LDL, mean ± SD, mmol/L	2.23 ± 0.64	2.60 ± 0.74	2.86 ± 0.78	<0.01	2.20 ± 0.65	2.47 ± 0.61	2.78 ± 0.77	<0.01
Cr, mean ± SD, μmol/L	64.24 ± 18.86	67.03 ± 25.24	68.17 ± 37.60	0.07	63.87 ± 50.22	63.76 ± 23.78	73.07 ± 59.24	0.04
BUN, mean ± SD, mmol/L	5.75 ± 1.98	5.96 ± 2.21	6.12 ± 2.94	0.05	6.22 ± 2.11	6.13 ± 2.04	6.66 ± 2.84	0.03
UA, mean ± SD, μmol/L	261.56 ± 78.20	274.96 ± 83.02	299.11 ± 101.10	<0.01	269.62 ± 84.76	285.79 ± 85.45	323.29 ± 94.81	<0.01
Triglycerides, mean ± SD, mg/dl	11.77 ± 3.05	18.74 ± 4.18	36.19 ± 22.09	<0.01	11.97 ± 3.25	18.68 ± 4.52	36.81 ± 24.58	<0.01
FBG, mean ± SD, mg/dl	99.03 ± 18.93	106.60 ± 23.91	134.41 ± 60.13	<0.01	96.24 ± 16.84	108.97 ± 29.17	131.77 ± 52.12	<0.01
Survival time, mean ± SD, days	1214.38 ± 570.51	1154.03 ± 553.25	1106.91 ± 579.31	0.01	1062.55 ± 555.85	1084.80 ± 559.20	1135.34 ± 556.59	0.32
All-cause death, N (%)				0.30				0.23
Survival	466 (91.19%)	471 (91.28%)	458 (88.76%)		224 (86.82%)	214 (84.58%)	227 (89.72%)	
Death	45 (8.81%)	45 (8.72%)	58 (11.24%)		34 (13.18%)	39 (15.42%)	26 (10.28%)	
Hypertension, N (%)				0.63				0.19
No	451 (88.26%)	445 (86.24%)	450 (87.21%)		223 (86.43%)	218 (86.17%)	230 (90.91%)	
Yes	60 (11.74%)	71 (13.76%)	66 (12.79%)		35 (13.57%)	35 (13.83%)	23 (9.09%)	
Diabetes, N (%)				0.50				0.18
No	490 (95.89%)	500 (96.90%)	493 (95.54%)		249 (96.51%)	244 (96.44%)	250 (98.81%)	
Yes	21 (4.11%)	16 (3.10%)	23 (4.46%)		9 (3.49%)	9 (3.56%)	3 (1.19%)	
Fracture classification, N (%)				0.79				0.19
Thoracic vertebra	94 (18.40%)	92 (17.83%)	96 (18.60%)		34 (13.18%)	37 (14.62%)	31 (12.25%)	
Lumbar vertebra	159 (31.12%)	153 (29.65%)	151 (29.26%)		64 (24.81%)	78 (30.83%)	78 (30.83%)	
Wrist	30 (5.87%)	47 (9.11%)	45 (8.72%)		27 (10.47%)	21 (8.30%)	16 (6.32%)	
Proximal humerus	73 (14.29%)	70 (13.57%)	69 (13.37%)		35 (13.57%)	17 (6.72%)	29 (11.46%)	
Femoral neck/trochanteric/subtrochanteric	155 (30.33%)	154 (29.84%)	155 (30.04%)		98 (37.98%)	100 (39.53%)	99 (39.13%)	

SD, standard deviation; BMI, body mass index; HDL, high-density lipoprotein; LDL, low-density lipoprotein; Cr, creatinine; BUN, blood urea nitrogen; UA, uric acid; FBG, fasting blood glucose.

### 3.2 Analysis of the association between ACM and TyG-I

**Figure 2:**
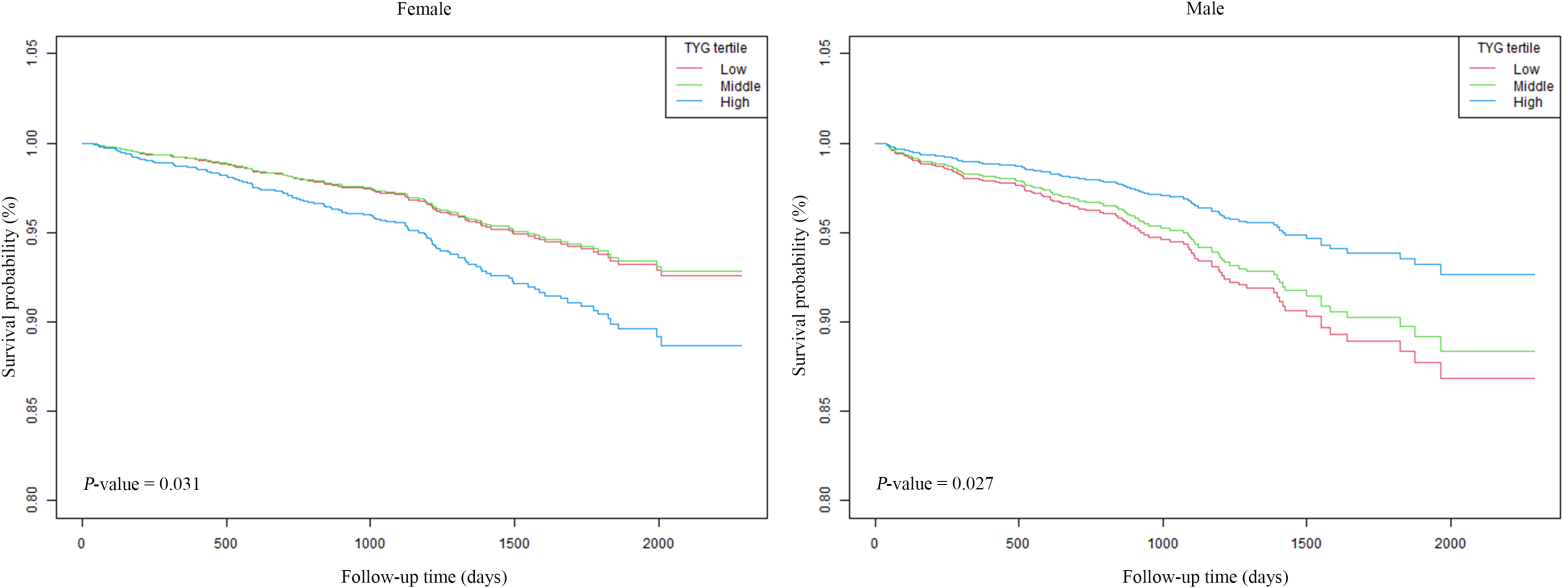
Kaplan–Meier curves for ACM stratified by TyG.

The associations between ACM and the TyG-I, analyzed separately by sex, are shown in **Table 2**. The hazard ratios (HRs) were evaluated in three distinct models for both female and male participants. In females, Model 1 (unadjusted) showed an HR for the TyG-I of 1.25 (95% CI: 0.98 to 1.59), with a *p-value* of 0.08. However, Model 3 (adjusted for age, BMI, hemoglobin, calcium, Cr, BUN, UA, and fracture classification) revealed a significant association, with an HR of 1.37 (95% CI: 1.06 to 1.77) and a *p-value* of 0.02. The highest TyG-I tertile in females was also related to a marked increase in risk in Model 3, yielding an HR of 1.67 (95% CI: 1.11 to 2.52) and a *p-value* of 0.01. For males, the TyG-I demonstrated an inverse association with ACM in Model 3, which adjusted for multiple variables, with an HR of 0.78 (95% CI: 0.54 to 1.12). However, the values were not statistically significant (*p =* 0.18). Notably, the highest tertile of TyG-I in males showed a significant reduction in risk in Model 3, with a HR of 0.53 (95% CI: 0.30 to 0.92) and a P-value of 0.02. Kaplan-Meier survival curves (**Figure 2**) were employed to elucidate the association of TyG-I tertiles with ACM. Among females, those in the highest TyG-I tertile had substantially higher ACM than those in the lowest tertile (*p =* 0.03). In contrast, males in the highest TyG-I tertile showed markedly lower ACM than the lowest tertile ones (*p =* 0.03).

**Table 2:** Associations of TyG-I and ACM in different sexes.

Sex	HR (95%CI) *P*-value		
	Model 1^a^	Model 2^b^	Model 3^c^
Female			
TyG	1.25 (0.98, 1.59) 0.08	1.24 (0.97, 1.59) 0.08	1.37 (1.06, 1.77) 0.02
TyG tertile			
Low	Reference	Reference	Reference
Middle	1.04 (0.69, 1.58) 0.84	0.95 (0.63, 1.43) 0.80	1.03 (0.68, 1.56) 0.90
High	1.41 (0.95, 2.08) 0.09	1.44 (0.97, 2.13) 0.07	1.67 (1.11, 2.52) 0.01
Male			
TyG	0.90 (0.65, 1.23) 0.50	0.83 (0.59, 1.17) 0.29	0.78 (0.54, 1.12) 0.18
TyG tertile			
Low	Reference	Reference	Reference
Middle	1.14 (0.72, 1.81) 0.57	0.91 (0.57, 1.44) 0.68	0.86 (0.53, 1.39) 0.54
High	0.73 (0.44, 1.22) 0.23	0.62 (0.37, 1.03) 0.07	0.53 (0.30, 0.92) 0.02

HR, hazard ratio; BMI, body mass index; Cr, creatinine; BUN, blood urea nitrogen; UA, uric acid.

a No adjustment.

b Adjusted for age and BMI.

c Adjusted for age, BMI, hemoglobin, calcium, Cr, BUN, UA, and fracture classification.

### 3.3 Restricted cubic spline analysis and threshold effects

**Figure 3:**
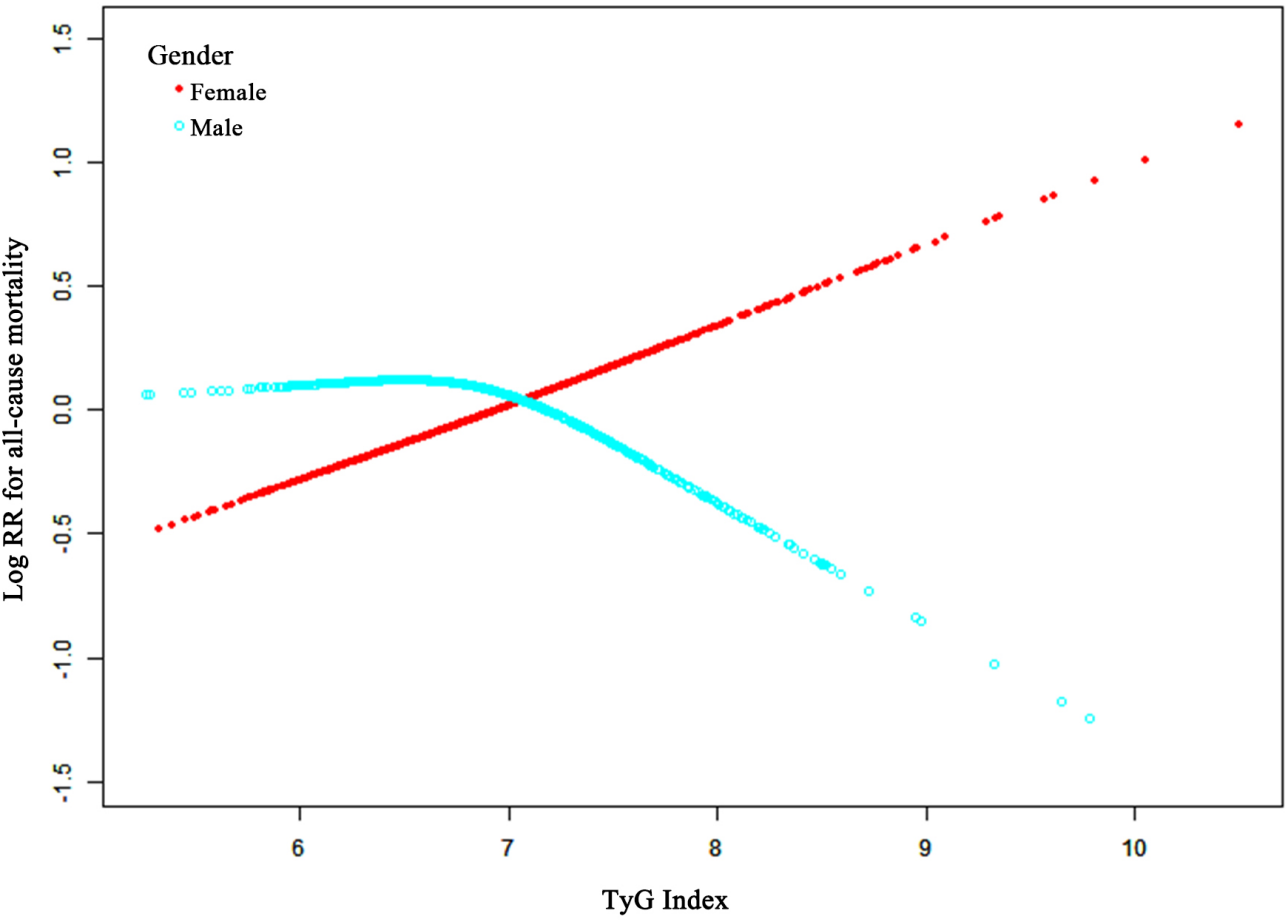
Adjusted smoothed curves illustrating the relationship between TyG-I and ACM risk after surgery for osteoporotic fractures in elderly patients. The red line represents females, and the cyan line represents males. The curves were adjusted for age, BMI, hemoglobin, calcium, creatinine, blood urea nitrogen, uric acid, and fracture classification. The y-axis shows ACM’s log relative risk (Log RR), and the x-axis represents the TyG-I values.

The association of the TyG-I with ACM was elucidated with restricted cubic splines for flexible modeling and visualization. Following adjustments for factors including age, BMI, hemoglobin, calcium, Cr, BUN, UA, and fracture classification, the TyG-I showed a linear correlation with ACM in female participants (**Figure 3**). Specifically, higher TyG-I values were associated with an increase in mortality risk in female participants. Furthermore, a significant interaction between sex and the TyG-I was detected (*p* = 0.02).

A two-stage linear regression model was applied to explore possible threshold effects, and the results are provided in **Supplementary Table S1**. This analysis revealed that a one-unit TyG-I increase was related to a 37% higher ACM risk in females (95% CI, 1.06 to 1.77; *p-value* = 0.02). However, no such relationship was found in male participants (HR, 0.78; 95% CI, 0.54 to 1.12; *p-value* = 0.18). For females, the two-segment nonlinear model (Model B) revealed a turning point for the TyG-I at 7.92. Below this value, the HR was 1.56 (95% CI: 1.11 to 2.20, *p =* 0.01), demonstrating a significant increase in mortality risk with an increasing TyG-I. Above this point, the HR decreased to 0.82 (95% CI: 0.31 to 2.18, *p =* 0.69), suggesting either a reduction in risk or stabilization. However, the likelihood ratio test comparing the linear and nonlinear models revealed no significant differences (*p >* 0.05). Analysis of these data revealed no advantage of the nonlinear model over the linear model in terms of model fit.

### 3.4 Interaction and sensitivity analyses

The findings of subgroup analyses demonstrating the association between ACM and the TyG-I across various population characteristics are summarized in **Table 3**. There was a statistically significant interaction between sex and the TyG-I (*p* = 0.01). Females showed a significantly elevated mortality risk (HR: 1.37, 95% CI: 1.06–1.77, *p =* 0.02), whereas the relationship in males was not statistically significant (HR: 0.78, 95% CI: 0.54–1.12, *p =* 0.18). For other subgroups, such as those based on age, BMI, fracture classification, Cr, BUN, UA, hemoglobin, and calcium levels, showed no significant interactions with TyG-I in relation to mortality risk (all *P* for interaction > 0.05).

**Table 3:** Subgroup analyses exploring the association between TyG-I and ACM.

Subgroup	no. of deaths/total no.	HR (95% CI)	*P*-value	*P*-value for interaction
Sex				0.01
Female	148/1543	1.37 (1.06, 1.77)	0.02	
Male	99/764	0.78 (0.54, 1.12)	0.18	
Age, years				0.77
50 - 62	4/747	2.24 (0.50, 10.09)	0.29	
63 - 72	28/756	0.94 (0.49, 1.81)	0.85	
73 - 100	215/804	1.11 (0.89, 1.40)	0.36	
BMI, kg/m^2^				0.51
<24	130/756	1.17 (0.91, 1.51)	0.22	
≥24,<28	60/782	0.88 (0.55, 1.42)	0.61	
≥28	57/769	1.04 (0.33, 3.25)	0.95	
Fracture classification				1.00
Thoracic vertebra	24/384	1.17 (0.51, 2.68)	0.70	
Lumbar vertebra	44/683	1.06 (0.61, 1.82)	0.85	
Wrist	4/186	0.00 (0.00, Inf)	0.99	
Proximal humerus	5/293	1.76 (0.15, 20.19)	0.65	
Femoral neck/trochanteric/subtrochanteric	170/761	1.08 (0.83, 1.39)	0.57	
Cr, μmol/L				0.26
10 - 54	65/755	0.84 (0.50, 1.43)	0.53	
55 - 68	82/773	1.33 (0.97, 1.83)	0.07	
69 - 863	100/779	1.01 (0.71, 1.44)	0.94	
BUN, mmol/L				0.36
1.55 - 4.98	81/752	1.33 (0.96, 1.84)	0.09	
5.00 - 6.49	72/785	1.00 (0.62, 1.61)	1.00	
6.50 - 52.80	94/770	0.89 (0.61, 1.29)	0.54	
UA, μmol/L				0.22
69 - 237	69/759	1.59 (0.98, 2.57)	0.06	
238 - 310	82/775	1.02 (0.69, 1.49)	0.93	
311 - 997	96/773	0.96 (0.69, 1.33)	0.80	
Hemoglobin, g/L				0.65
43.0 - 119.9	75/755	0.95 (0.61, 1.50)	0.84	
120.0 - 133.9	87/748	0.98 (0.62, 1.56)	0.95	
134.0 – 177.0	86/788	1.17 (0.87, 1.57)	0.31	
Calcium, mmol/L				0.05
1.47 - 2.14	74/715	1.07 (0.70, 1.64)	0.76	
2.15 - 2.25	87/811	1.46 (1.02, 2.10)	0.04	
2.26 - 2.91	86/781	0.79 (0.54, 1.13)	0.20	

Adjusted for age, sex, BMI, fracture classification, Cr, BUN, SUA, Hemoglobin, and Calcium, except the subgroup variable.

HR, hazard ratio; BMI, body mass index; Cr, creatinine; BUN, blood urea nitrogen; UA, uric acid.

## 4 Discussion

This research retrospectively elucidated the association of the TyG-I with ACM in 2,307 patients admitted for osteoporotic fractures requiring surgical intervention. Adjustments were made for confounding factors, including BMI, hemoglobin, age, calcium levels, Cr, BUN, UA, and fracture classification. In female patients, each unit increase in the TyG-I was linked with a 37% rise in ACM risk. These results indicate that TyG-I can serve as an important metabolic health indicator in women with osteoporosis. However, there was no significant relation observed in male patients. Paradoxically, male patients in the highest TyG-I tertile exhibited a 47% reduction in mortality risk (HR = 0.53), suggesting that sex-specific biological mechanisms may underlie this relationship.

IR is defined as a reduced response to both endogenous and exogenous insulin, impairing glucose uptake and metabolism in insulin-targeted organs (21). This condition is often related to elevated serum TG levels and decreased HDL cholesterol (HDL-c) concentrations (22, 23). Based on these concepts, the TyG-I, which uses fasting TG and glucose measurements, has emerged as a reliable biomarker for detecting IR. Research demonstrates that TyG-I provides higher accuracy in assessing IR compared to the standard HOMA-IR method (24, 25). Research has frequently shown that the TyG-I is positively linked with mortality risk in diabetes or CVD patients. These findings indicate that elevated levels of the TyG-I are correlated to a higher risk of death, particularly in conditions driven by metabolic abnormalities. Retrospective cohort studies by Chen et al. and Li et al. found a statistically significant increase in both all-cause and cardiovascular mortality among individuals with higher TyG indices (7, 26). Similarly, in patients suffering from chronic coronary syndrome or ischemic stroke, an increased TyG-I was related to an elevated ACM risk (27, 28). Nevertheless, some studies have questioned the connection between the TyG-I and overall mortality. For instance, a meta-analysis involving 6,354,990 participants found no significant link between the TyG-I and ACM in the general population (8). Another cohort study by Vega showed that the correlation of the TyG-I with mortality risk became statistically non-significant after adjusting for factors such as age, BMI, resting systolic blood pressure, and smoking habits (29). The observed heterogeneity among these studies may result from the small sample of included studies and the relatively short follow-up periods, potentially limiting statistical power. Therefore, additional research studies are required to establish the association of the TyG-I with ACM. This study offers new and important perspectives on the role of the TyG-I in various populations and extends its applicability. However, the relationship between the TyG-I and mortality has been extensively explored in the context of CVD and diabetes. This investigation studied the previously under-explored association of the TyG-I with ACM in patients with OPFs, revealing significant correlations, particularly in females.

The relationship between the TyG-I and OPFs remains underexplored. Previous research from our group has suggested that a higher TyG-I negatively impacts bone turnover, potentially accelerating osteoporosis progression (30). In the current study on hospitalized OPF patients, it was observed that the TyG-I serves as an essential independent risk factor for increased mortality. Although the TyG-I is widely recognized for its strong predictive value in the development and progression of diseases, the biological mechanisms underlying its effects are not yet fully elucidated. The association between the TyG-I and ACM highlights the potential role of IR. Moreover, IR plays a critical role in the development of diabetes and is closely associated with a higher risk of CVD. Among these, atherosclerosis-related cardiovascular conditions are a leading cause of global morbidity and mortality (31). The underlying biological processes are complex, with IR serving as a central factor (32, 33). Meta-analyses and extensive research have consistently highlighted the strong association between IR and atherosclerotic CVD (34–37).

Under normal physiological conditions, insulin interacts with cell membrane receptors to initiate a signaling cascade that promotes glucose uptake and activates nitric oxide (38), a powerful vasodilator and anti-atherosclerotic molecule (39). However, during IR, this signaling pathway becomes dysfunctional (40). It leads to impaired glucose metabolism and potentially contributes to elevated blood pressure and the development of atherosclerosis. In response to IR, pancreatic β-cells enhance insulin secretion to overcome the resistance (41, 42). At elevated levels, insulin functions as a strong growth factor, activating the Mitogen-Activated Protein Kinase (MAPK) pathway, which drives vascular smooth muscle cell proliferation and differentiation (43). This process also enhances inflammation and activates transcription factors contributing to vascular smooth muscle proliferation and atherosclerotic plaque development (44, 45). As a result, compensatory hyperinsulinemia not only fails to restore normal insulin signaling but may also accelerate atherosclerosis progression.

Sex differences may serve as an important factor influencing this association. The I-Lan Longitudinal Aging Study (ILAS) found sex-related variations in the relationship between increased TyG indices and subclinical atherosclerosis in individuals without diabetes. Among non-diabetic women, those with high TyG indices had a significantly higher prevalence of subclinical atherosclerosis than those with low TyG indices (OR = 1.510; 95% CI 1.010 to 2.257), while no such association was observed in men (OR = 0.827; 95% CI 0.556 to 1.231) (11). This pattern aligns with our finding of increased mortality risk in females with high TyG-I.

Multiple physiological and metabolic factors may explain these sex-specific variations. First, fundamental differences exist in fat distribution patterns between sexes that influence the development of insulin resistance (IR) and diabetes (46, 47). Men typically accumulate more visceral and ectopic fat (48), whereas women store fat subcutaneously (49). Since visceral fat is markedly linked with IR, women need to gain more weight and show more significant deterioration in metabolic risk factors to accumulate similar visceral fat levels as men (50). Second, hormonal factors contribute substantially to these differences, with estrogen providing protective effects against insulin resistance until menopause, as demonstrated by Tramunt et al. (51). This aligns with research on bone fragility, which shows significant sex-specific metabolic profiles, particularly in diabetic patients. Metabolomic analyses have revealed that while women’s bone fragility is heavily influenced by hormonal stages and pregnancy, men present a more homogeneous model for studying metabolic disruptions in bone metabolism, with distinct alterations in acylcarnitines and glycerophospholipids (52). After menopause, women may experience accelerated metabolic deterioration. Third, research by Kautzky-Willer et al. (53) indicates that women with diabetes experience disproportionately higher risk of cardiovascular complications compared to men with similar glycemic control. Finally, Parks et al. (54) identified sex-specific gene expression patterns related to insulin signaling that could explain differential responses to metabolic stress. These biological differences collectively suggest that metabolic risk factors become evident differently across sexes as individuals transition from normoglycemia to elevated glucose levels and diabetes, supporting our conclusion that TyG-I is positively correlated with all-cause mortality risk specifically in female patients, while exhibiting different relationships in males.

When elevated, the TyG-I may influence ACM by enhancing systemic inflammatory responses. It has been observed that IR significantly elevates systemic inflammation (55), which profoundly affects osteoporosis and bone healing (56). This chronic inflammation disrupts metabolic health and directly affects bone metabolism, leading to potential detriments in bone structure and function (57). Furthermore, IR is typically characterized by higher levels of inflammatory markers, such as tumor necrosis factor-alpha (TNF-α), C-reactive protein (CRP), and interleukin-6 (IL-6), which play a direct role in regulating osteoclast and osteoblast activity. In the presence of inflammation, TNF-α and IL-6 activate osteoclasts, enhancing their bone-resorbing activity while simultaneously inhibiting the bone-forming functions of osteoblasts (58, 59). Bone healing involves stages such as inflammation, cartilage formation, bone formation, and remodeling. Although inflammation is vital in the early stages of healing, persistent inflammation due to IR can disrupt this process, resulting in excessive inflammation, suppression of bone formation, and impaired healing. This disruption leads to delayed or incomplete bone healing in individuals with osteoporosis and fractures, thus increasing the risk of mortality. Therefore, managing IR and its associated inflammation is critical. Reducing systemic inflammation can facilitate better bone healing, prevent complications, and lower mortality rates.

The results of this study hold considerable clinical significance. First, the TyG-I offers a practical, low-cost approach to identifying high-risk patients. Specifically, triglycerides and fasting glucose, the components used to calculate the TyG-I, can be easily measured through routine blood tests. The TyG-I can also be readily calculated during standard preoperative assessments, which include evaluations of liver and kidney function and electrolyte levels for patients undergoing surgery for osteoporotic fractures. In routine clinical settings, regularly monitoring the TyG-I can enhance the ability to identify high-risk patients, especially women with osteoporosis. Including this index in clinical management can optimize care strategies by adjusting treatment plans, refining pharmacological therapies, and improving follow-up procedures to reduce mortality risk. Furthermore, the study emphasizes the need to address metabolic health in caring for patients with osteoporotic fractures. The TyG-I reflects IR, and elevated levels may indicate metabolic disruptions related to an increased risk of CVD and diabetes. Therefore, for patients with higher TyG levels, treatment should extend beyond conventional osteoporosis management to address related metabolic conditions, such as improving insulin sensitivity and adjusting lipid metabolism. Further, the study’s observation of gender differences highlights the importance of considering sex-specific factors in future research and clinical practice. Treatment and intervention strategies may need to be customized to the distinct physiological and metabolic needs of each sex to achieve the best outcomes.

This study possesses several strengths. Initially, it addresses an important gap in the current literature. To the best of our knowledge, the association of the TyG-I with ACM in hospitalized patients undergoing surgical procedures for OPF has not been previously explored. The TyG-I offers significant potential for clinical application in the management of OPF patients. The study’s extended follow-up period and sufficient endpoint events ensured robust statistical power, allowing for a detailed observation of mortality rates. The open enrollment design adopted in this study facilitated the inclusion of diverse populations, thereby improving the external validity of the findings and minimizing selection bias.

Despite the valuable insights provided by this study, several limitations must be acknowledged. First, being observational, it does not establish a cause-and-effect relationship between TyG indices and ACM. Second, the analysis was limited to baseline TyG indices, and the potential effects of changes in these indices during the follow-up period on ACM remain uncertain and require further research. Lastly, since the study was conducted at a single institution with a relatively small cohort, the results may not be generalizable to other ethnic groups. To ensure the consistency and broader relevance of these results, more extensive studies are needed, including multi-center randomized trials, the incorporation of additional biomarkers, and investigations involving diverse ethnic groups.

## 5 Conclusions

A significant sex-specific association between the TyG-I and ACM was observed in patients with osteoporotic fractures. For female patients, an increase of one unit in the TyG-I was associated with a 37% higher mortality risk. In contrast, male patients in the highest tertile of the TyG-I demonstrated a reduced mortality risk. The TyG-I is potentially a valuable prognostic marker, particularly for female patients. Its established reliability, simplicity, and cost-effectiveness make it suitable for inclusion in routine clinical assessments. These results emphasize the need for sex-specific care in managing osteoporotic fractures and the consideration of metabolic health in treatment approaches.

## Data Availability

The original contributions presented in the study are included in the article/**Supplementary Material**. Further inquiries can be directed to the corresponding authors.
