# Supplementary material for: Sex-specific association between triglyceride-glucose index and all-cause mortality in patients with osteoporotic fractures: a retrospective cohort study
Source: Front Endocrinol (Lausanne). 2025 Apr 30;16:1574238. doi: 10.3389/fendo.2025.1574238 (PMC12074978; doi:10.3389/fendo.2025.1574238)
Supplement: Supplementary file 2 [file Table1.docx]

Table S1 Threshold analyses examining the relationship between TyG index and all-cause mortality

|  | Model 3^a^ | | | *P*-value for interaction^e^ |
| --- | --- | --- | --- | --- |
|  | Female | Male | Total |  |
|  | HR (95% CI) *P*-value | HR (95% CI) *P*-value | HR (95% CI) *P*-value |  |
| Model A^b^ |  |  |  | 0.02 |
| One line effect | 1.37 (1.06, 1.77) 0.02 | 0.78 (0.54, 1.12) 0.18 | 1.10 (0.89, 1.37) 0.37 |  |
| Model B^c^ |  |  |  | 0.06 |
| TyG turning point (K) | 7.92 | 6.50 | 7.76 |  |
| < K | 1.56 (1.11, 2.20) 0.011 | 1.85 (0.40, 8.57) 0.430 | 1.18 (0.89, 1.56) 0.254 |  |
| > K | 0.82 (0.31, 2.18) 0.687 | 0.65 (0.40, 1.06) 0.082 | 0.85 (0.39, 1.86) 0.685 |  |
| Slope 2-Slope 1 | 0.52 (0.17, 1.64) 0.27 | 0.35 (0.06, 2.05) 0.25 | 0.72 (0.29, 1.82) 0.49 |  |
| LRT^d^ | 0.24 | 0.23 | 0.48 |  |

HR hazard ratio, CI confidence interval, BMI body mass index, Cr creatinine, BUN blood urea nitrogen, UA uric acid, LRT logarithmic likelihood ratio test

^a^Adjusted for age, BMI, hemoglobin, calcium, Cr, BUN, UA, and fracture classification

^b^Linear analysis, *P*-value < 0.05 indicates a linear relationship

^c^Nonlinear analysis

^d^*P*-value < 0.05 means Model B is significantly different from Model A, which indicates a nonlinear relationship

^e^*P*-value for the sex-interaction, P-interaction<0.05 indicates a stronger interaction effect
